# Admission Time and Surgical Technique are Important in Aortic Dissection Treatment Results

**DOI:** 10.21470/1678-9741-2020-0085

**Published:** 2020

**Authors:** Mesut Engin

**Affiliations:** 1 Department of Cardiovascular Surgery, University of Health Sciences, Mehmet Akif İnan Training and Research Hospital, Şanlıurfa, Turkey. mesut_kvc_cor@hotmail.com

To the Editor,

I have read the article by Bedel et al.^[1]^, entitled “Association of Platelet to Lymphocyte and Neutrophil to Lymphocyte Ratios with In-Hospital Mortality in Patients with Type A Acute Aortic Dissection” with great interest. First of all, I congratulate the authors for their invaluable contribution to the literature. However, I would like to clarify some points about the aortic dissections.

Aortic dissections are diseases with higher mortality and morbidity rates among cardiovascular diseases. Many factors play a role in the success of treatment. The most important are the surgical technique, the brain protection method used, the patient's condition of malperfusion at the time of admission to the clinic, and the time between surgery and the first symptom^[2]^. In addition, in case of type A dissection, only the ascending aorta limited dissection status (DeBakey type 2) may also have an impact on mortality.

Routine blood parameters have been extensively studied in cardiovascular diseases. The most important of them stands out as neutrophil-lymphocyte ratio (NLR). In the study by Öz et al.^[3]^, the relationship between NLR and type A aortic dissection mortality was investigated. In the multivariate analysis performed in this study, in addition to NLR, surgical-related factors, such as additional surgical procedures and cardiopulmonary bypass time, were also identified as independent predictors of mortality. In another similar study, the effects of surgical factors on mortality, other than NLR, have also been shown^[4]^.

However, in this current study investigating the relationship between blood parameters and type A dissection mortality, no surgical details were given. As far as we understand from the study, 10 patients died before they could be operated on. Total surgery was performed in 86 patients and mortality occurred in 7 patients who underwent surgery. However, postoperative mortality and mortality without surgery were evaluated in the same group and the relationship between blood parameters were investigated^[1]^. As we mentioned in the studies above^[3,4]^, it may be misleading to evaluate mortality only with reference blood parameters when surgery is applied to patients with aortic dissection. In addition, the most important assessment to be made here for emergency specialists should be the time from the first symptom to hospital admission. NLR may also have increased due to increased adventitial pressure and intimal ischemia during this time. It is known that ischemic events can increase NLR^[5]^.

In addition, low platelet count may indicate false lumen size. The large false lumen is associated with poor results. Accordingly, there are publications in the literature showing that low platelet count may be associated with poor results^[6]^. Therefore, establishing a relationship between preoperative platelet-lymphocyte ratio (PLR) and mortality can also provide misleading results.

Finally, in the multivariate analysis, the authors identified age, white blood cell count, neutrophil count, lymphocyte count, NLR, PLR and surgery as independent predictors of mortality. Are the authors sure that factors other than age and surgery are independent predictors according to the 95% confidence interval (CI) values given? I think there might be a typo in the 95% CI values given.
